# Using qualitative risk assessment to re-evaluate the veterinary fence paradigm within the Kavango Zambezi Transfrontier Conservation Area

**DOI:** 10.3389/fvets.2025.1702631

**Published:** 2026-01-30

**Authors:** Laura E. Rosen, Shirley J. Atkinson, Nlingisisi D. Babayani, Mokganedi Mokopasetso, Mary-Louise Penrith, Nidhi Ramsden, Janine Sharpe, Thompson Shuro, Odireleng I. Thololwane, Jacques van Rooyen, Steven A. Osofsky

**Affiliations:** 1 Victoria Falls Wildlife Trust, Victoria Falls, Zimbabwe; 2 AHEAD Program, Cornell University College of Veterinary Medicine, Ithaca, NY, United States; 3 Okavango Research Institute, University of Botswana, Maun, Botswana; 4 Botswana Vaccine Institute, Gaborone, Botswana; 5 Department of Veterinary Tropical Diseases, Faculty of Veterinary Science, University of Pretoria, Onderstepoort, South Africa; 6 Seanama Conservation Consultancy, Gaborone, Botswana; 7 Directorate of Scientific Services, Ministry of Environment, Forestry and Tourism, Windhoek, Namibia; 8 Directorate of Veterinary Services, Ministry of Agriculture, Fisheries, Water and Land Reform, Windhoek, Namibia; 9 Department of Veterinary Services, Ministry of Lands and Agriculture, Gaborone, Botswana; 10 REHerd Africa (Pty) Ltd, George, South Africa; 11 Centre for Sustainable Agriculture, Department of Sustainable Food Systems and Development, Faculty of Natural and Agricultural Sciences, University of the Free State, Bloemfontein, South Africa

**Keywords:** African buffalo, contagious bovine pleuropneumonia, foot and mouth disease, habitat connectivity, risk analysis, transboundary animal diseases, veterinary fences

## Abstract

**Introduction:**

Habitat connectivity in southern Africa’s Kavango Zambezi Transfrontier Conservation Area (KAZA TFCA, or KAZA) is hindered by the presence of veterinary fences put in place to prevent transboundary animal disease transmission. In northern Botswana’s Ngamiland, much of the fencing infrastructure is in disrepair due to ineffective maintenance in the face of increased elephant damage, but specific sections of some fences still restrict critical wildlife movements.

**Methods:**

We undertook qualitative risk assessments for sections of the Northern Buffalo fence near the Okavango Delta and the Zambezi Border and Western Border fences along the Botswana-Namibia borders. We assessed multiple risk pathways for three main transboundary animal diseases (foot and mouth disease, contagious bovine pleuropneumonia and peste des petits ruminants) under three different scenarios: (1) the status quo (fences as they currently are), (2) with hypothetical removal of specific fence sections, and (3) with hypothetical removal of fence sections with risk mitigation measures instituted.

**Results:**

Our study found that hypothetical removal of these fence sections did not increase the risk of the transboundary animal diseases of interest, and that with the institution of specific risk mitigation measures (such as strategic livestock herding), the overall risk of some diseases would be lower compared to the status quo. Each pathway contained critical steps with low, very low or negligible risk which influenced the overall risk for the pathway.

**Discussion:**

Based on low estimated risks, sections of all three fences could be considered for removal, but further information was needed for the Western Border fence. Key stakeholders established consensus to move forward with consultations with local communities and to offer assistance with the implementation of risk mitigation measures (such as improved herding, kraaling) conditionally associated with potential removal of key fence sections. Opening the fences in key low-risk areas would restore connectivity for elephants and other wildlife and potentially reduce human-wildlife conflict in areas where high densities of elephants are constrained by fences. This new, more sectorally integrative approach to livestock disease control is vital to wildlife’s ability to access key resources over space and time and thus to the sustained success of KAZA.

## 1 Introduction

**Figure 1:**
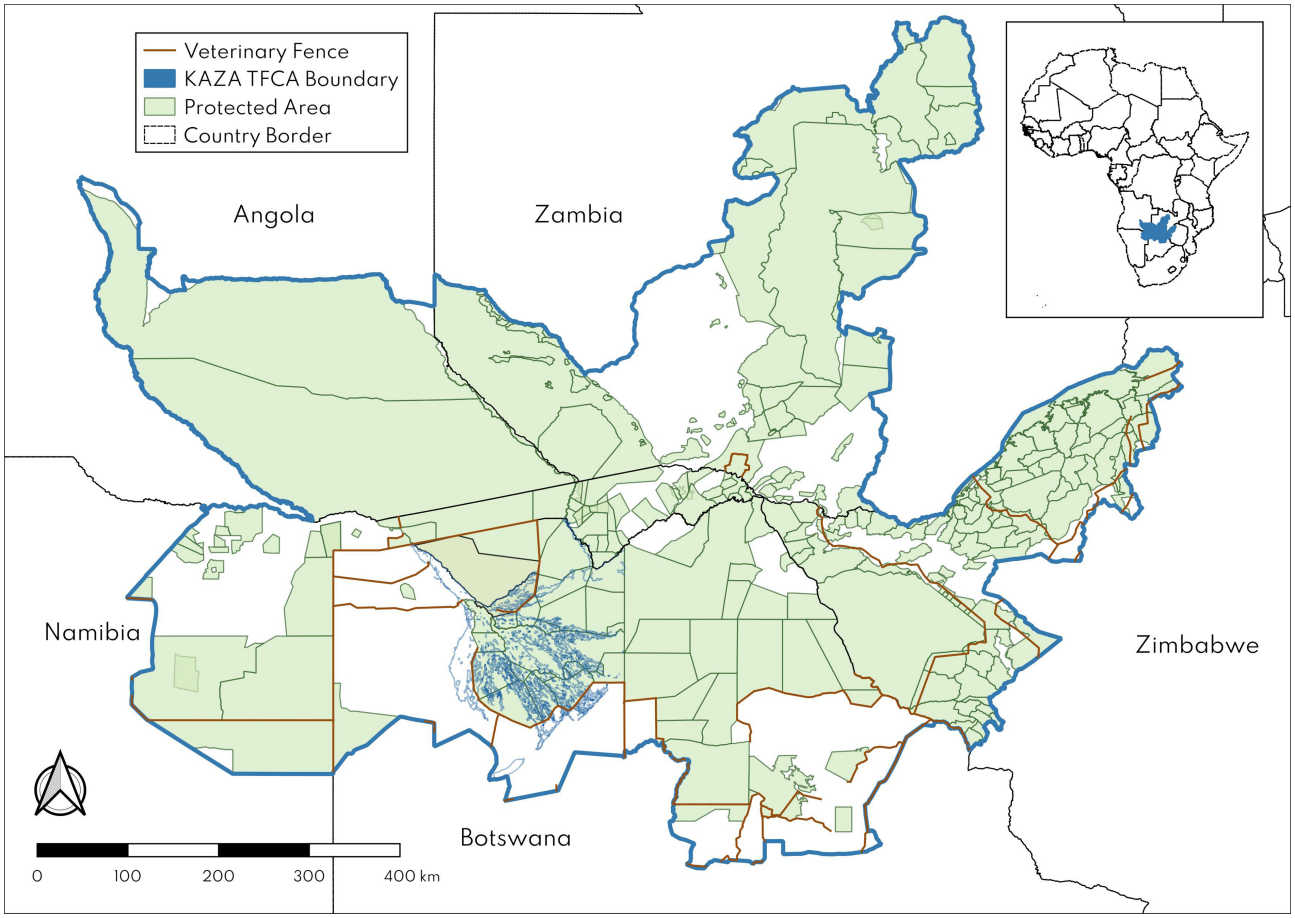
The Kavango Zambezi (KAZA) Transfrontier Conservation Area (TFCA) in southern Africa and existing fences, in brown, within the landscape. Green polygons represent different types of protected areas within the TFCA. Inset map shows extent of KAZA TFCA in southern Africa. The KAZA TFCA shapefile was used with permission from the Peace Parks Foundation.

The Kavango Zambezi (KAZA) Transfrontier Conservation Area (TFCA), hereafter also referred to as KAZA, is the largest terrestrial TFCA in the world, encompassing more than 520,000 km^2^ of Angola, Botswana, Namibia, Zambia and Zimbabwe. It is home to extraordinary biodiversity across unique ecosystems, including the Okavango Delta, and supports the largest remaining population of African savanna elephants (*Loxodonta africana*), estimated at ~228,000 in a 2022 survey (1). At the core of the 2011 KAZA Treaty is an emphasis on securing connectivity of habitats critical for wildlife and thus ensuring intactness of the region’s key migratory routes (2). A stark challenge to this goal is the presence of numerous fences that cut across the KAZA landscape (Figure 1), inhibiting wildlife movements since construction began in the late 1950s (3). In many cases, these fences were erected to protect against the threat of transboundary animal diseases such as foot and mouth disease (FMD) and contagious bovine pleuropneumonia (CBPP). FMD in particular has driven the construction of fences to limit contact between cattle and African buffalo (*Syncerus caffer*), which are a reservoir host for the South African Territories (SAT) serotypes of FMD virus endemic in southern Africa.

FMD is considered among the most economically important animal diseases in the world because of its significant trade implications for countries or zones not free of the disease (4). In southern Africa, veterinary fences have played a key role in the World Organisation for Animal Health (WOAH) standards for geographic control of FMD, focused on eradicating the disease from a prescribed zone or country which can then be considered free of FMD. In Botswana and Namibia, communal farmers living in “red” or infected zones – areas where buffalo are usually present and FMD is endemic – have long been marginalized without access to the more profitable international beef export markets (e.g., European Union) available to farmers living in “green” zones (those free of FMD) (5). Notably, cattle morbidity and mortality observed in outbreaks of endemic SAT-type FMD tend to be low compared to what is seen with other strains circulating elsewhere in the world (6), but communal farmers have experienced great hardship during FMD outbreaks, when cattle movements and slaughter were prohibited over large areas and sometimes for long periods of time. New opportunities to address this problem arose with the advent of commodity-based trade (CBT), which focuses on ensuring that beef products have negligible risk of harboring FMD virus (FMDV) by ensuring biosafety along the beef value chain, even if cattle originate from areas where FMD is endemic (7). CBT thus launched new possibilities for export of beef without the need for fences to separate cattle from buffalo (7).

Northwestern Botswana’s Ngamiland is one such area where communal farmers have experienced increasing FMD outbreaks since 2007. A study of Ngamiland households found that 73% reported hardship due to lack of cattle sales and 89% stated a need for alternative livelihoods (8). This mirrors larger trends in Botswana over the last 60 years, where the agriculture sector’s contribution to GDP has declined from 40% to 2% while the tourism sector – reliant on Botswana’s wildlife – has surged to 11.5% of GDP (9). Fewer adults choose to engage in herding – a low paying, socially undesirable occupation – over other livelihood options, and children who previously tended cattle now attend school instead (10). The lack of incentives to better manage livestock and subsequent reduced availability of herders have resulted in households increasingly leaving their cattle to roam unattended, resulting in rangeland degradation around villages and fewer cattle presented for mandatory scheduled official vaccinations such as against FMDV (8). Inadequate herding (and associated kraaling at night) also contributes to higher levels of livestock loss to predators and makes stock theft easier.

Veterinary fencing’s contribution to disease prevention in the region may also be declining as the dynamics of FMD evolve. Buffalo have historically been the most important source of FMDV to cattle in southern Africa, but outbreaks in Ngamiland over the last 20 years mostly occurred ≥55 km from the Okavango Delta where buffalo are present, while no outbreaks occurred in many areas adjacent to the Delta (11, 12). In East Africa, multiple studies provide evidence for independent circulation of different strains of FMDV in buffalo and cattle (13–15). Ol Pejeta Conservancy in Kenya holds 8,500 cattle and 1,200 buffalo in continual spatial and temporal contact through shared grazing and water points, but a study there found no evidence of buffalo-cattle FMDV transmission and noted that cattle in the conservancy experience very few clinical outbreaks of FMD compared to cattle in the surrounding communities which have far less buffalo exposure (13). It is possible that SAT-type FMDV is occasionally introduced into cattle populations from buffalo contact, but that these strains have low morbidity and mortality, and in combination with routine vaccination against FMDV, may subsequently circulate endemically among cattle with only periodic episodes of clinical disease (12, 16). In such a situation, veterinary fences do not offer protection against outbreaks from strains already circulating in the cattle population.

Some of the existing veterinary fences in Ngamiland were erected to control CBPP, a disease of cattle with no wildlife involvement, rather than FMD. Botswana had been free of CBPP for over 50 years when it recurred in 1994 and spread through Ngamiland (17). CBPP has a potential chronic carrier state and vaccination is only 33–67% effective at 3 months post-vaccination (18), making it challenging to eradicate. Fences were constructed to try to contain the outbreak but failed to do so, and ultimately all ~320,000 cattle in the district were stamped out (17). The cost of this eradication effort was estimated at BWP 360,850,000 (US$97.5 million) (19). The CBPP cull also prompted an exodus of labor from Ngamiland’s agriculture sector, as the eradication of all cattle forced former herders to seek out alternative livelihoods, and many did not return to herding after restocking began (20). Although CBPP is not zoonotic, the aftermath of how this outbreak was managed revealed profound human health impacts. The lack of meat and milk from cattle resulted in a 35% increase in childhood malnutrition at the household level in the affected area, and one third of 12–23 month old children in Ngamiland were severely underweight in the following years (21).

Looking back, southern Africa’s veterinary fencing paradigm offers an important cautionary tale from a One Health perspective (22). The principle of veterinary fencing to separate populations of animals with differing disease status is intuitive, but fences have historically been constructed without being informed by environmental impact assessments or community engagement, with varying impacts for local communities and wildlife populations (22). Members of the multi-ethnic population across the Okavango Delta and northwestern Ngamiland do not share a uniform perspective on preferred livelihood options, and exhibit a broad spectrum of opinions on land-use priorities as well as on veterinary fences (23, 24). For some subsistence livestock farmers, fences limit accessible grazing and water points for cattle (23). These limitations are echoed in their effects on wildlife, hundreds of thousands if not millions of which have died from entanglements and the inability to access critical food and water sources during the dry season, with numerous species in the Delta having experienced significant population declines (22, 25, 26). Diminished populations of large herbivores represent a risk to the valuable wildlife-viewing tourism industry in the Okavango Delta (27, 28). In western Ngamiland, such losses of wild herbivores may have led to greater accumulation of plant biomass, fueling more intensive wildfires (20).

**Figure 2:**
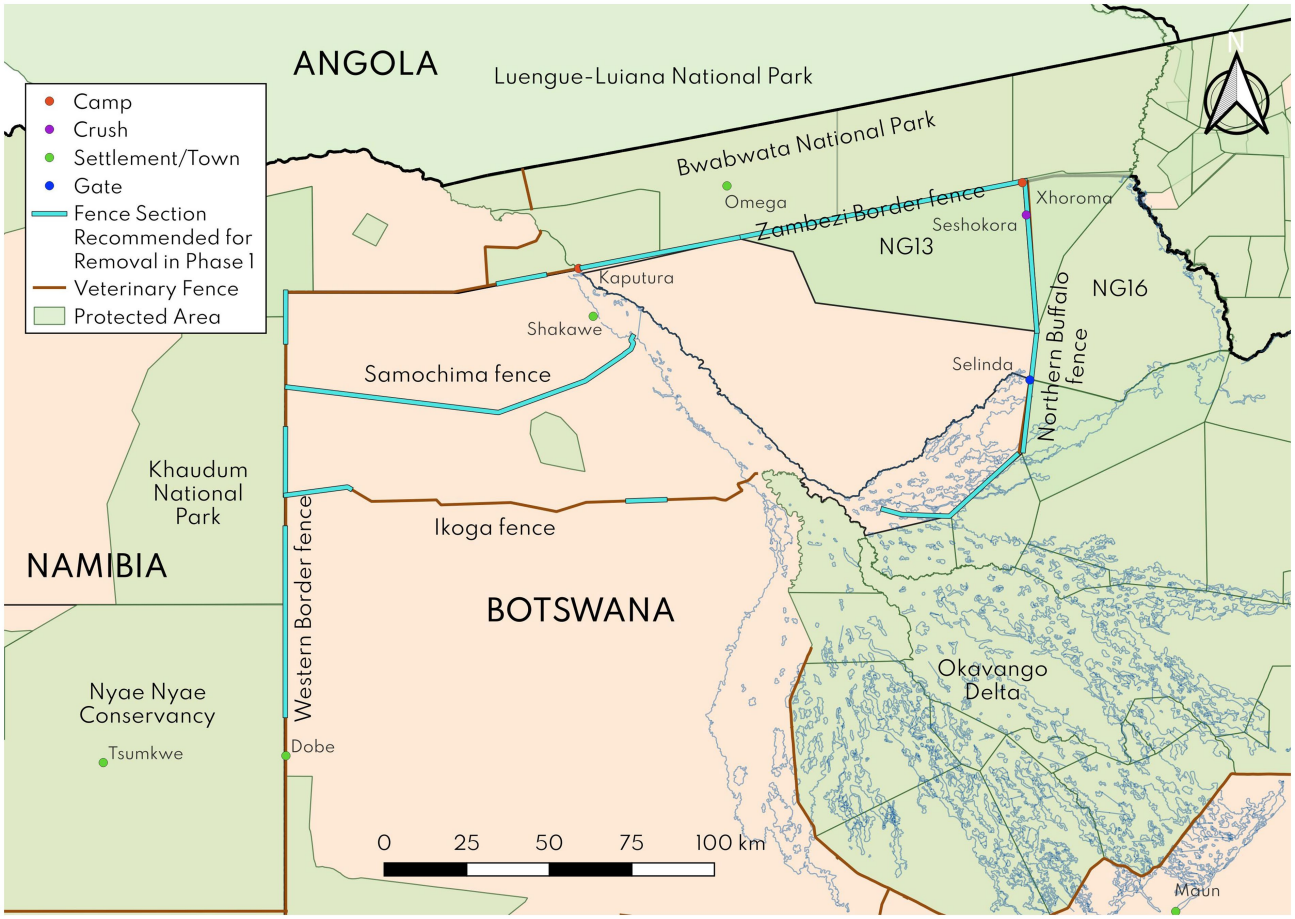
Map of veterinary fences in Ngamiland, Botswana and selected settlements, camps, crushes and gates. The blue fence sections represent fence sections recommended for removal from a wildlife conservation perspective in the Phase 1 study. Note that the Samochima and Ikoga fences were not evaluated in the Phase 2 analysis, but they are still important to consider due to their impacts on wildlife movements, as per the Phase 1 analysis findings.

Elephant numbers in the eastern panhandle of the Okavango Delta, on the other hand, have ballooned to about 18,000 – one of the highest elephant densities in KAZA (1). This population is constrained by the Okavango River and the Zambezi Border (formerly Caprivi) and Northern Buffalo fences (see Figure 2), which restrict movement (particularly for matriarchal herds with calves, as explained below) out of the panhandle into the Kwando River Wildlife Dispersal Area (29). Human-elephant conflict in the panhandle has been on the rise, with elephants destroying crops and causing human fatalities (30). Elephant radio collar data paint a compelling picture of some males, but not females, occasionally crossing these and other fences in Botswana (31). Naidoo et al. (31) speculate that this may be due to the inability of calves to cross fences; the veterinary fences in question have a steel cable at 1.1 m which often remains intact even when fence wire strands are broken, and this height appears to block elephant calves from crossing either under or over. Elephant cows are capable of crossing but will not leave their calves behind, therefore entire cow-calf herds remain effectively trapped within the panhandle.

In the face of a growing elephant population and limited resources to sustain maintenance of thousands of kilometers of fences, as well as an acknowledgement of significant environmental impacts antithetical to the stated vision of KAZA as a connected landscape supporting both biodiversity conservation and poverty alleviation (16), a variety of stakeholders in KAZA have come together to reconsider the extent of veterinary fence deployment (32). Fences bring a host of cross-sectoral complications in TFCAs like KAZA, and critically, fences only work as intended when they remain intact. Fences under challenge by elephants must be electrified to prevent damage and require continuous monitoring and repair. Those that are not maintained may be dismantled by communities seeking to access grazing or water resources on the other side, or the materials may be repurposed for securing livestock elsewhere or made into poaching snares (33). In some locations across KAZA, periodic flooding also damages fencing. Veterinary departments do not have the financial or human resources to maintain fences across thousands of kilometers, nor is it clear that such a level of subsidization of the livestock sector currently makes economic sense (22).

History from within KAZA shows that removing fences can restore the connectivity that is central to the TFCA’s goals. The Nxai Pan veterinary fence in Botswana, erected in 1968 (3), was removed in 2004, and there was evidence of renewed wildlife movements within a year (34) and by 2008, long-distance migration of zebra occurred along a migratory path that had been blocked for decades (35). Similar evidence of wildlife movement restoration was seen with the 2003 removal of Botswana’s Setata fence (34), originally constructed in 1995 (3). A 30 km section of the Zambezi Border fence [originally the Caprivi fence, constructed in 1996 (3)] was removed in 2001 (creating the “Kwando Corridor”) (36), and elephant collar data from 2010 onwards showed that elephants moved freely through this area again (31).

The KAZA Animal Health Sub Working Group identified formal assessment of veterinary fences as a priority activity in its workplan in 2019. The Animal Health Sub Working Group, an official body under KAZA, serves to improve animal health and mitigate disease and related conflicts within the TFCA. It includes senior representatives from the departments of veterinary services, animal production and wildlife from each of the KAZA partner states, as well as from regional and international non-governmental organizations (including WOAH and the Food and Agriculture Organization). In 2020, the group initiated an envisioned three-phase study: Phase 1 would evaluate which fence sections in Botswana were most negatively impacting wildlife movements (29), Phase 2 would evaluate how the risks of important livestock diseases might change, or not, if those fence sections were removed (32), and Phase 3 (currently underway) would evaluate the perspectives of local communities regarding specific fence sections that might be considered for potential removal, as well as explore communities’ potential interests in strategic herding. The overall goal is that the three phases of work will help inform national, bilateral and KAZA-level planning efforts within the context of science-based regional collaboration in the areas of disease risk management, natural resource use and management, and community development (32).

Herein we provide an overview of the Phase 2 study (32), the objective of which was to compare the risks of transboundary animal diseases (FMD, CBPP, PPR) in portions of KAZA under the status quo versus hypothetical scenarios of removal of specific fence sections in Botswana (with and without institution of risk mitigation measures).

## 2 Materials and methods

### 2.1 Development of core team and objectives

We selected sections of three fences that were identified in the Phase 1 report (29) as high priorities for potential removal based on their impacts on wildlife movements (Figure 2). These were (i) a ~ 80 km section of the Northern Buffalo fence within Ngamiland, from its juncture with the Zambezi Border fence southwards, (ii) a ~ 90 km section of the Zambezi Border fence, east of the Okavango River, and (iii) three sections of the Western Border fence, which measure ~15, ~20 and ~60 km, north of Dobe border post. The Northern Buffalo fence is entirely internal to Botswana and separates livestock rearing areas as well as a wildlife management area (NG13) from other wildlife areas with buffalo. The Zambezi Border fence section separates Bwabwata National Park in Namibia from NG13 as well as livestock rearing areas in Botswana. The Western Border fence sections separate Khaudum National Park and Nyae Nyae Conservancy in Namibia from livestock rearing areas in Botswana.

Our risk assessments for each fence line focused on comparing three different scenarios for each disease of interest: the status quo (with fences in their current condition), a hypothetical scenario of removing the fence sections above, and a hypothetical scenario of removing these fence sections and instituting risk mitigation measures. The status quo scenario served to establish the baseline level of risk with fences under current conditions, which was not zero. The fence removal scenario served to demonstrate the effect of fence removal itself. The removal and risk mitigation scenario offered a more nuanced portrait of how fence removal might be undertaken with proactive risk management.

We first compiled a review of methodologies for risk assessment which was circulated among the core team of collaborators in veterinary services and wildlife departments from the Governments of Botswana and Namibia and regional experts in epidemiology, infectious disease control, and livestock management in September 2022. We held initial consultative meetings with the Government of Botswana in September 2022. Based on the limitations of data available, we agreed on using the WOAH guidelines for qualitative import risk analysis (37), with a focus on hazards of FMD and CBPP in cattle. We included both endemic SAT-type FMD and FMD serotype O, which has emerged in Zambia and Namibia in recent years (38). FMDV serotype O is considered a serious threat given its greater morbidity and mortality compared to SAT-type FMDV infections and the fact that most cattle in the region are not vaccinated against it.

**Figure 3:**
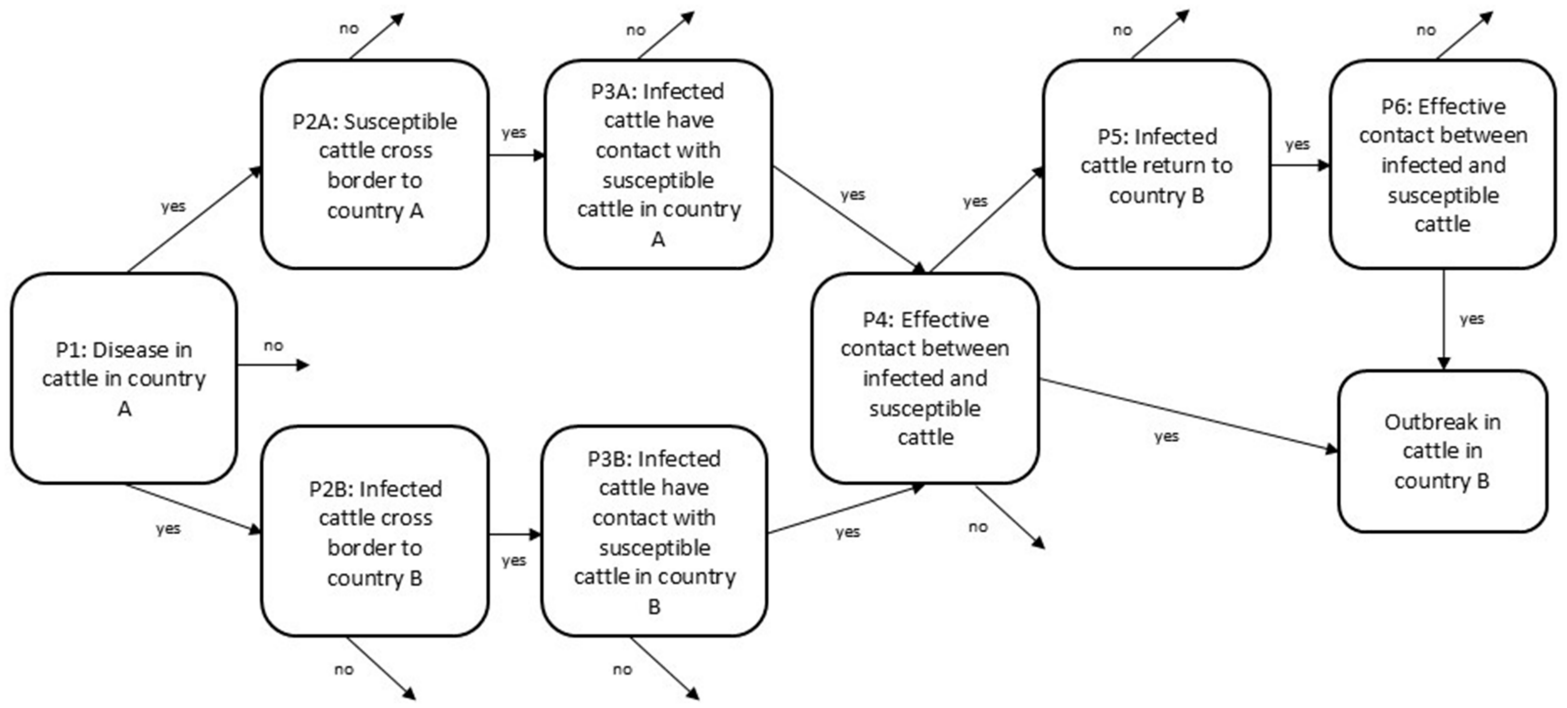
Generic risk pathway for foot and mouth disease and contagious bovine pleuropneumonia via cattle-cattle contact. Pathways for cattle-buffalo contact followed the same general pattern.

**Figure 4:**
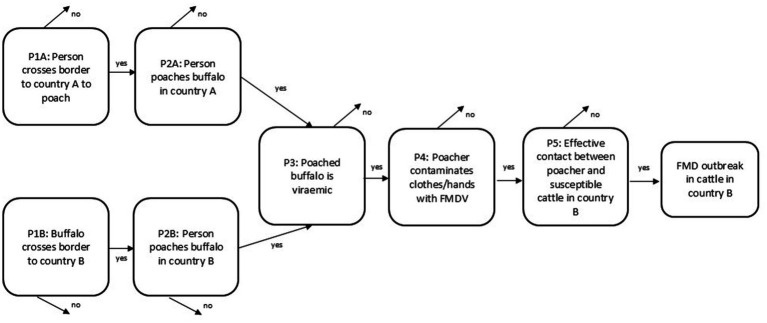
Generic risk pathway for foot and mouth disease via poaching.

We developed risk pathways for the FMD and CBPP hazards and modified them during a virtual discussion among key collaborators in February 2023 (generic pathways shown in Figures 3, 4). We included three pathways for SAT-type FMDV transmission to cattle: from cattle, from buffalo and from poachers (a hypothesized pathway for outbreaks in Botswana which had not been researched but which was of particular concern to some government veterinarians). We did not include buffalo or poaching risk pathways for FMD serotype O as there is no evidence of buffalo maintaining FMDV serotype O in the wild nor isolation of FMDV serotype O from buffalo (32). Because CBPP is strictly a disease of cattle, with no evidence of infection in African buffalo or other wildlife species (39), we only assessed the risk of cattle-cattle transmission. The pathways for each disease and fence line are shown in Table 1.

**Table 1:** Summary of disease risk pathways considered at each fence section of interest in the risk assessment.

Disease	Northern Buffalo fence	Zambezi Border fence	Western Border fence
SAT-type FMD	Buffalo in zone 16 to cattle in zone 2 Poacher in zone 16 to cattle in zone 2	Cattle in Namibia to cattle in Botswana Buffalo in Namibia to cattle in Botswana Poacher in Namibia to cattle in Botswana Cattle in Botswana to cattle in Namibia Buffalo in Botswana to cattle in Namibia Poacher in Botswana to cattle in Namibia	Cattle in Namibia to cattle in Botswana Buffalo in Namibia to cattle in Botswana Poacher in Namibia to cattle in Botswana Cattle in Botswana to cattle in Namibia Buffalo in Botswana to cattle in Namibia Poacher in Botswana to cattle in Namibia
Serotype O FMD	N/A	Cattle in Namibia to cattle in Botswana	Cattle in Namibia to cattle in Botswana
CBPP	N/A	Cattle in Namibia to cattle in Botswana	Cattle in Namibia to cattle in Botswana
PPR	N/A	Small ruminants in Namibia to small ruminants in Botswana	Small ruminants in Namibia to small ruminants in Botswana

We added peste des petits ruminants (PPR), a disease of concern for small ruminants, as a third transboundary animal disease of interest after further discussions with Botswana and Namibia officials. Notably, PPR has never been confirmed in Namibia and the southern portion of the country holds PPR-free status, but its neighbors Angola and Zambia border countries where PPR is endemic (40).

### 2.2 Data collection

We reviewed literature on FMD, CBPP, PPR and impacts of fencing, and collated data from WOAH’s World Animal Health Information System on FMD, CBPP and PPR outbreaks in the region. We downloaded audit reports from the European Commission Directorate-General for Health and Food Safety for the Botswana and Namibia animal health control systems and a 2018 report on control of FMD by Botswana Department of Veterinary Services (DVS) from the Botswana Office of the Auditor General. We compiled digital and hard copy records from Botswana DVS, Namibia Directorate of Veterinary Services and Botswana Vaccine Institute. These included vaccination records, surveillance data, post-vaccination monitoring results, strain sequencing data and maps of outbreaks and crushes. Crushes are community-level facilities used for handling livestock for inspections, treatments and vaccinations, and represent the nearby herds served by the facilities, as well as the smallest epidemiological unit for Botswana DVS disease control. Okavango Research Institute provided questionnaire data from surveys conducted with farmers and security agents near the veterinary fences of interest. We downloaded 2020–2030 Namibia Ministry of Environment, Forestry and Tourism Management Plans for Bwabwata and Khaudum National Parks, as well as 2019 aerial surveys of Khaudum and Zambezi Region conducted by the Ministry, a 2012 risk analysis of animal disease hazards associated with import of animal commodities into Namibia, and the 2022 KAZA Elephant Survey reports. Cattle numbers and locations in Nyae Nyae Conservancy, location data from collared cattle, and aerial survey data on presence of wildlife and livestock in the eastern panhandle were shared by local NGOs. We discussed perceived poaching risk, fence conditions, animal movements, and disease risk with government and conservation NGO employees working near the fences of interest.

We reviewed hard copy records from the Botswana DVS FMD Unit in Maun, Botswana in November 2022. Following the visit to Maun, we visited the DVS office in Shakawe, Botswana and conducted a one-day drive covering ~140 km of the Zambezi Border fence from Kaputura Camp (a small DVS facility housing field staff) near the Okavango River to Xhoroma Camp (a former DVS facility now occupied by the Botswana Defence Force) at the far eastern end of the fence, then proceeded south for 62 km of the Northern Buffalo fence to Selinda Gate. We noted the fence conditions along the drive with photos and videos.

### 2.3 Hazard identification

We identified three transboundary animal diseases as hazards of interest for our assessment. These included FMDV serotypes SAT-1, SAT-2 and SAT-3 of the *Aphthovirus* genus in family *Picornaviridae*, causing FMD in cattle, as hazards to both Botswana and Namibia. We also assessed the risk of FMDV serotype O, but only from cattle in Namibia to cattle in Botswana as FMDV serotype O has not been reported from Botswana. We assessed the risk of *Mycoplasma mycoides* subsp. *mycoides* small colony variant (MmmSC), endemic in parts of southern Africa and causing CBPP in cattle. Botswana holds official WOAH CBPP-free status, therefore we only assessed the risk of MmmSC from cattle in Namibia to cattle in Botswana. Finally, we assessed the risk of peste des petits ruminants virus of the *Morbillivirus* genus in family *Paramyxoviridae*, causing PPR in small ruminants. We only assessed the risk of PPR from small ruminants in Namibia to small ruminants in Botswana because Botswana holds country-wide PPR-free status from WOAH. Namibia holds PPR-free status for the zone south of the Veterinary Cordon Fence (or “Red Line”), where commercial farms are located; the communal cattle farming area north of the Red Line has no official PPR status but has never had evidence of PPR infection.

### 2.4 Risk assessment

We assessed the risk and uncertainty for a given pathogen at each step in the risk pathways for each fence (generalized pathways shown in Figures 3, 4; detailed pathways shown in original report (32)). Risks were assigned one of five categories (Supplementary Table 1), ranging from negligible to high, adapted from Rinchen et al. (41). Uncertainty was ranked as low, moderate, or high (defined in Supplementary Table 2) as described by Fournié et al. (42). We also assessed the general consequences of a disease outbreak for each pathogen and each country (defined in Supplementary Table 3), using criteria from Zepeda-Sein (43). We used a combination matrix (Supplementary Table 4) modified from that used by Rinchen et al. (41) to calculate entry and exposure risks. This matrix structure accounts for the fact that probabilities exist between 0 and 1 and therefore, when multiplying probabilities, the product cannot be higher than the lower probability (44). The lowest probability levels in the pathway can therefore function as “risk bottlenecks” that limit the overall risk of the pathway. We combined the entry and exposure risks using the same matrix to calculate an overall risk of probability of occurrence for the pathway.

We used a conservative approach for uncertainty in which the highest level of uncertainty from the pathway was retained (45). We used a separate risk matrix (Supplementary Table 5) to combine the *probability of occurrence* and the *consequences* for an overall risk estimate. This risk matrix, adapted from Dufour et al. (46), weights the overall risk based on the severity of the consequences. This system aligns well with how transboundary animal disease risk is often interpreted in the region, in that if the probability of a disease being introduced is small but the consequences could be devastating, the overall risk is considered greater than just the small risk of introduction.

For scenarios of fence removal with risk mitigation, we considered the following risk mitigation measures: vaccination, post-vaccination monitoring, removal of cattle from Bwabwata National Park, increased security patrols, and the REHerd model of strategic active herding and kraaling which has been implemented in pilot sites in KAZA (32). Vaccination against FMD and CBPP is practiced to varying degrees in some areas of KAZA, depending on disease status, and post-vaccination monitoring, when undertaken, helps to ensure that animals are adequately protected. Removal of cattle from Bwabwata National Park relates to the fact that Namibian law mandates that no cattle should be present in national parks, but enforcement of this law has been stalled by complex sociopolitical barriers (47). Increased security patrols relate to areas along international borders and anti-poaching patrols along the buffalo fence. Under the REHerd model, cattle are actively herded during the day and kraaled in a predator-proof boma at night, ideally in a mobile kraal, with a planned grazing regime. Cattle are also branded and/or have appropriate identification to allow traceability, and trained herders maintain records for all cattle, including vaccinations. Herders avoid contact with wildlife (particularly buffalo, impala (*Aepyceros melampus*) and predators) and with cattle outside their combined herd. For the purposes of this assessment, compliance with these practices is assumed to be high but not perfect, and this is accounted for in the characterization of the degree of uncertainty for assessments wherein the REHerd model is applied.

### 2.5 Risk assessment validation

We compiled the draft risk assessment report and circulated it to the key collaborators in April 2024. Members of the core team met for a validation meeting on the report over two and a half days in Maun in May 2024. During the meeting, we reviewed four representative pathways in detail and recorded comments to be clarified in the final report. We divided the attendees into breakout groups for the Government of Botswana, the Government of Namibia and non-state experts and asked them to provide feedback on the report recommendations, which were then presented to the entire group. The group agreed on revised recommendations, and we revised the report and recommendations based on comments from the meeting and completed the final draft in July 2024. We circulated this report a final time to the co-authors for any final suggestions, and published it online in November 2024 (32).

## 3 Results

Below we present a brief summary of fence conditions and, for each disease, the assessed probability of occurrence, consequences and overall risk estimate at each fence, under all three fencing scenarios [(1) the status quo (fences as they currently are), (2) with hypothetical removal of specific fence sections, and (3) with removal of fence sections along with institution of risk mitigation measures]. Given that we conducted 20 assessments, we only present the “risk bottlenecks” in each pathway that limit the overall risk rather than every step from every pathway. We also present the recommendations for each fence from the validation meeting. Detailed consequence assessments, explanations of proposed risk mitigation measures, descriptions of the risk for each step in each pathway under each scenario, and risk matrix calculations are found in our original report (32).

### 3.1 Northern Buffalo fence

On our visit to the Northern Buffalo fence, we found the fence condition was generally good close to Xhoroma Camp, with the fence largely upright and intact. Further south its condition deteriorated, with missing wires, cable and gum poles noted. We noted some large gaps with all fencing material gone, including the cable. The fence has not been cleared of vegetation for years, with considerable growth in some places. Aerial survey data (32) and the Phase 1 surveys (29) documented the presence of buffalo in the eastern panhandle (on the “wrong” side of the fence), although they were largely concentrated at the southernmost aspect of the fence which was not proposed for removal.

The probability of occurrence and risk estimates for this fence are summarized in Table 2. The overall probability of occurrence for SAT-type FMD from buffalo was *very low* with moderate uncertainty under all three scenarios based on the very low risk (moderate uncertainty) of effective buffalo-cattle contact. Even in experimental studies with co-housed cattle and buffalo, the animals avoided one another and were unlikely to have direct contact (48). Buffalo can clearly transmit FMDV to cattle (49, 50), but the mechanism is not well understood and FMD outbreaks only occur sporadically even in areas where buffalo and cattle are in close proximity. Chobe District in Botswana, in comparison to Ngamiland, has no fences separating buffalo and cattle but has higher vaccination coverage and more active herding and kraaling practices due to an appreciation of the risk from predators, and has had one FMD outbreak event reported to WOAH in the last decade, while Ngamiland has had seven. In a study where susceptible cattle were herded with FMD infected buffalo, transmission of FMDV only occurred between buffalo and not to cattle (51). In another, cattle did not become infected with FMDV after sharing drinking troughs and hay racks with infected buffalo (52).

**Table 2:** Northern Buffalo fence risk scenarios summary of findings.

Northern Buffalo fence			
Risk scenario Disease/Route/Country	Status quo	Fence removal	Fence removal with risk mitigation
	Probability of occurrence/risk estimate		
SAT-FMD/buffalo/Botswana	Very low/Low	Very low/Low	Very low/Low
SAT-FMD/poaching/Botswana	Negligible/Low	Negligible/Low	Negligible/Low

Note that for SAT-type FMD outbreaks caused by a strain not covered by the vaccine, the overall risk estimate is moderate.

The probability of occurrence for SAT-type FMD from poaching was *negligible* with moderate or high uncertainty under all scenarios. This was driven by the very low risk (low uncertainty) of an adult buffalo being viremic and the negligible risk (moderate uncertainty) of effective contact between a poacher (a hypothetical fomite) and cattle; neither risk changed with fence removal. Natural infection with FMDV occurs around 3–6 months when buffalo calves, not an ideal target for bushmeat poachers hunting large game, lose their maternal immunity, and buffalo in the region are almost all serologically positive for SAT-type FMDV antibodies by age 2 (53). The period of viraemia in calves is short, with some studies suggesting 1–3 days (48) and other risk assessments using 3–5 days (54) or up to 14 days, although the risk of virus excretion in adults was considered negligible in that study (55). Effective contact between a poacher and cattle was also considered negligible. A quantitative risk assessment of routes of livestock exposure to FMDV from contaminated meat in the UK estimated that the risk of infection from human fomites, such as contamination of hands followed by handling livestock, was <0.01% of the total risk from all pathways (56).

The consequences of a SAT-type FMD outbreak were ranked *moderate* with low uncertainty. Although the morbidity and mortality associated with SAT-type FMD are typically low, there are indirect effects on milk yield and draught performance (57) as well as significant costs associated with control. In the event that an outbreak strain is not covered by the vaccines that have been in use, the virus would be expected to have greater morbidity and spread, and therefore consequences would be *high* with low uncertainty. For the Northern Buffalo fence under all scenarios, the overall risk estimate for SAT-type FMD from buffalo is *low* with moderate uncertainty and from poaching is *low* with moderate to high uncertainty.

At the validation meeting, we recommended the northern 62 km of the fence, from Xhoroma camp to Selinda gate, be considered for removal pending implementation of two risk mitigation measures. These were implementation of the REHerd model in villages in the eastern panhandle and resettlement of the cattle at Seshokora, a single isolated crush near the northern part of the fence (or implementation of REHerd practices for these cattle). Eastern panhandle communities are critical stakeholders in terms of potential buy-in and implementation of recommendations offered, thus consultations are required (Phase 3 of the program of work, as described above).

### 3.2 Zambezi Border fence

On our visit to the Zambezi Border fence east of the Okavango River, we found the fence conditions to be highly variable, from completely intact and upright to lying on the ground with missing wires. Conditions worsened away from Kaputura Camp, where some maintenance is still performed by Botswana DVS staff within 5 km of the camp. The top two strands of wire were missing in many places and many of the gum poles and droppers have rotted or completely disintegrated. We observed downed fence sections of up to 100 m. There has been no bush clearing on most of the fence and trees up to 3–4 m were growing near and through the fence.

**Figure 5:**
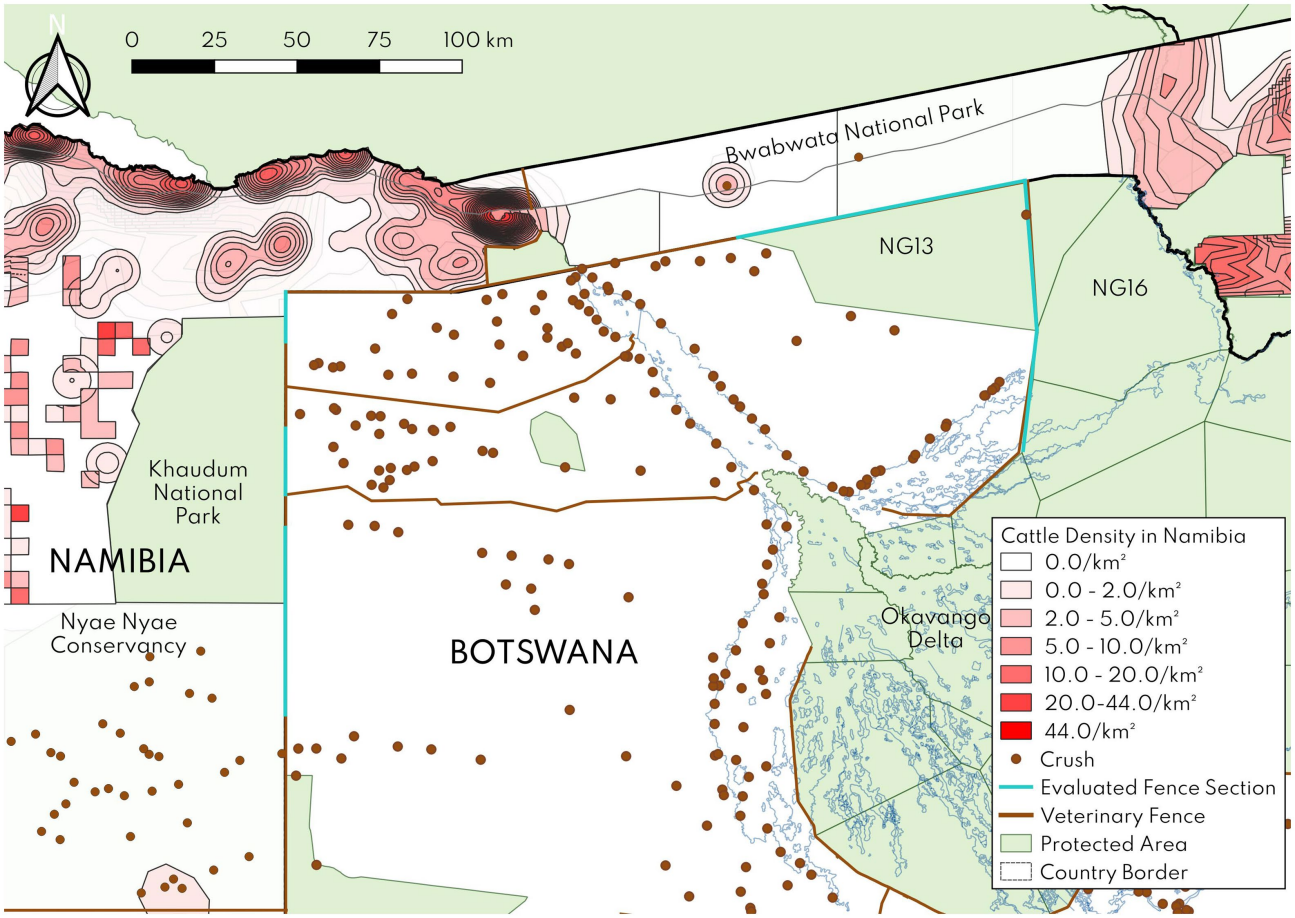
Map of Ngamiland, Botswana fence sections of focus for the disease risk assessment (light blue), with available crush data shown. Crush geodata were not available for Namibia so only select villages and publicly available cattle density data (2012 data for former Kavango Region around Khaudum National Park, 2002 Atlas of Namibia Project data otherwise) are plotted to give an indication of where cattle are most prevalent.

The probability of occurrence and risk estimates for this fence are summarized in Table 3. The probability of occurrence for SAT-type and serotype O FMD from Namibian cattle to cattle in Botswana under the status quo and fence removal scenarios were *very low* with moderate uncertainty, owing to the very low risk (moderate uncertainty) of either cattle crossing from Bwabwata National Park into Botswana or cattle from Botswana traversing the park and having contact with the cattle there. Cattle in Bwabwata are centered around Omega settlement (Figure 5), which is ~15 km from the Zambezi Border fence. The high density of predators and lack of surface water in this area of the park during the dry season make it inhospitable for cattle to move long distances. FMDV-infected cattle in the maximal shedding stage would also be less able to walk long distances. On the Botswana side of the fence, the section proposed for removal has a low density of cattle posts, and those closest to Omega are still ~20 km away (Figure 5). The probability of occurrence for SAT-type FMD from buffalo was also *very low*, owing to the very low risk (moderate uncertainty) of effective contact between buffalo and cattle as described in the Northern Buffalo fence section. The probability of occurrence for SAT-type FMD from poaching was *negligible* with moderate–high uncertainty under all scenarios, as described above.

**Table 3:** Zambezi Border fence (east of Okavango River) risk scenarios summary of findings.

Zambezi Border fence			
Risk scenario Disease/Route/Country	Status quo	Fence removal	Fence removal with risk mitigation
	Probability of occurrence/risk estimate		
SAT-FMD/cattle/Botswana	Very low/Low	Very low/Low	Negligible/Low
SAT-FMD/buffalo/Botswana	Very low/Low	Very low/Low	Very low/Low
SAT-FMD/poaching/Botswana	Negligible/Low	Negligible/Low	Negligible/Low
FMD type O/cattle/Botswana	Very low/Moderate	Very low/Moderate	Negligible/Moderate
SAT-FMD/cattle/Namibia	Very low/Low	Very low/Low	Negligible/Low
SAT-FMD/buffalo/Namibia	Very low/Low	Very low/Low	Negligible/Low
SAT-FMD/poaching/Namibia	Negligible/Low	Negligible/Low	Negligible/Low
CBPP/cattle/Botswana	Very low/Moderate	Very low/Moderate	Negligible/Moderate
PPR/small ruminants/Botswana	Very low/Low	Very low/Low	Very low/Low

Note that for SAT-type FMD outbreaks caused by a strain not covered by the vaccine, the overall risk estimate is moderate.

The probability of occurrence for FMD dropped to *negligible* under the fence removal with risk mitigation scenario, dependent on removal of cattle from Bwabwata National Park. Removing the cattle population from the park virtually eliminates the risk of either disease being transmitted across the border there.

The consequences for SAT-type FMD are *moderate,* as described above, therefore the overall risk estimate for SAT-type FMD was *low* with moderate or high uncertainty under all three scenarios. Animals that have never been exposed to or vaccinated against FMDV serotype O typically experience severe clinical signs (57), with higher apparent morbidity rates than for SAT-type outbreaks (6). Control costs are also higher for outbreaks of FMD serotype O if cattle are vaccinated with both the standard trivalent SAT-type vaccine and the serotype O vaccine. As a result, the consequences of FMD serotype O are considered *high* with low uncertainty. The overall risk estimate for FMD serotype O was *moderate* with moderate uncertainty under all three scenarios.

The probability of occurrence for CBPP was also *very low* under the status quo and fence removal scenarios due to the very low risk (moderate uncertainty) of cattle movement described above and the very low risk (moderate uncertainty) of CBPP in cattle in the former Caprivi strip area of Namibia (now Kavango East and Zambezi Regions). The last CBPP outbreak in Zambezi Region occurred in 2003, and outbreaks in Kavango East in the last decade have been small and located far from Bwabwata National Park.

Infection with MmmSC typically causes severe clinical signs and high mortality in naïve herds, with losses up to 80% (39). An outbreak of CBPP would result in Botswana losing its official CBPP-free status. Botswana does not have a contingency plan outlining how it would respond to a CBPP outbreak; it would require a lengthy process to regain country-wide free status from WOAH. Assuming stamping out were used again, the consequences are considered *high* with low uncertainty. The overall risk estimate was therefore *moderate* with moderate uncertainty.

The probability of occurrence for PPR was *very low* with low or moderate uncertainty under all scenarios, given very low (low–moderate uncertainty) entry and very low (low uncertainty) exposure risks. PPR has not been recorded in Namibia nor anywhere within hundreds of kilometers of the fence. Even if it were present, small stock have a more limited ability to walk long distances compared to cattle and would be highly unlikely to traverse the remote NG13 wildlife area to areas along the eastern panhandle where small stock are kept. Illegal movement of small stock poses the greatest risk for entry of PPR, which is likely not related to fence status, similar to the poaching scenarios. The consequences of a PPR outbreak were considered *moderate* with moderate uncertainty based on morbidity and mortality, trade implications and meat availability. The overall risk estimate across all scenarios was *low* with low–moderate uncertainty.

**Figure 6:**
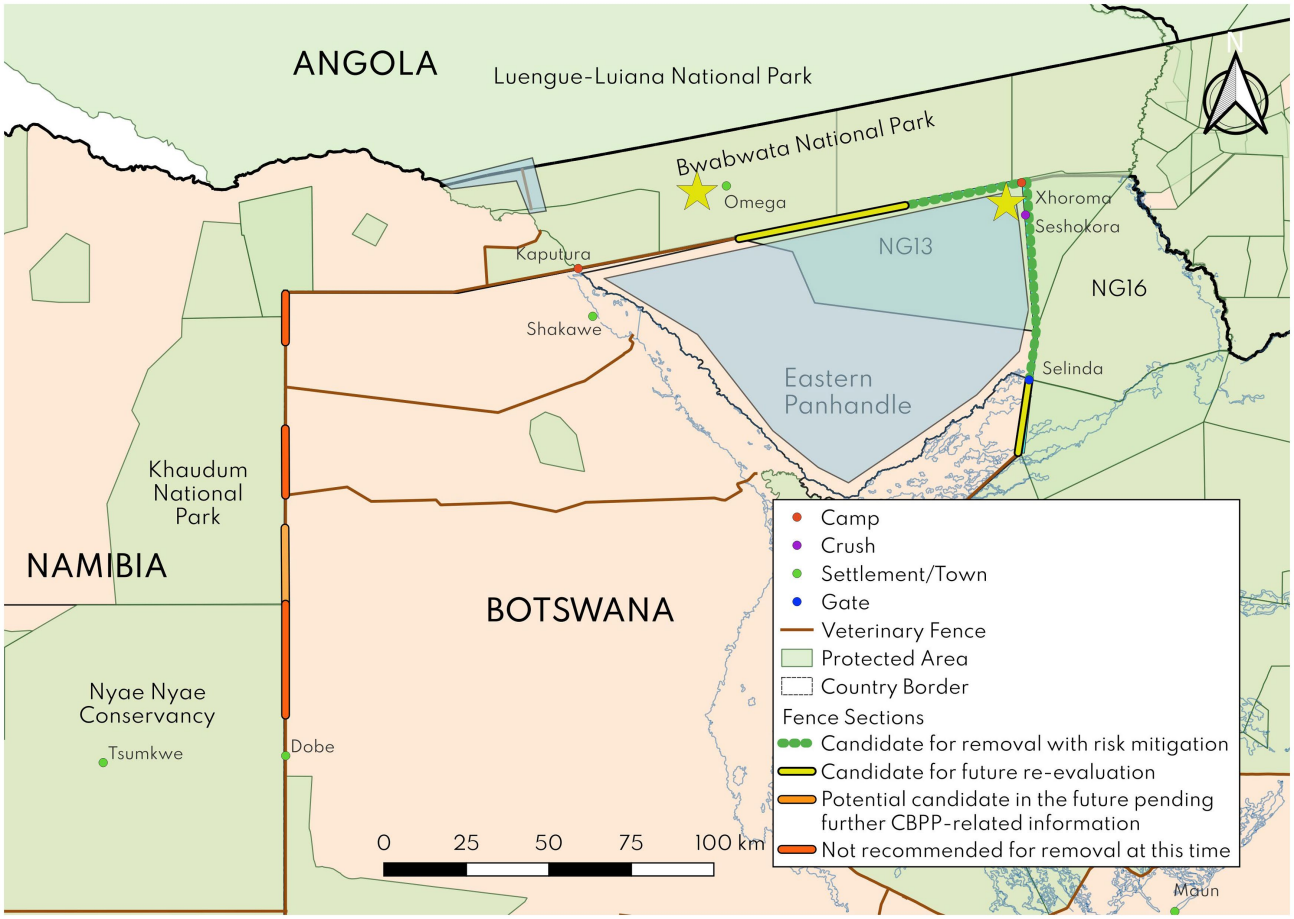
Map of final recommendations from the risk assessment. Fence sections recommended for community consultations are shown in green. Yellow stars (YS) and blue polygons (BP) indicate areas for risk-mitigation implementation: (BP) fencing of the far northwestern section of Bwabwata National Park; (YS) removal of cattle from the Omega settlement area; (YS) resettlement of the farmer at Seshokora crush or participation in REHerd herding practices; and (BP) participation in REHerd herding practices across villages in the eastern panhandle.

At the validation meeting, we agreed that the eastern 35 km of the fence (Figure 6) could be considered for potential removal pending community consultations and implementation of risk mitigation measures. These measures were to fence the western multiple use area of Bwabwata National Park (specifically the Namibia-Angola border and to repair the buffalo fence on the eastern side) and to remove the cattle from the multiple resource use area of the park near Omega settlement.

### 3.3 Western Border fence

We were unable to visit the Western Border fence for this assessment and gauged conditions based on fence patrol reports and the Phase 1 report. Photos provided by a colleague who visited the fence in March 2024 (32) confirmed similar conditions to what we observed at the other fences, with variable conditions, some large breakages and an elephant actively crossing from Namibia into Botswana.

The probability of occurrence and risk estimates for this fence are summarized in Table 4. The probability of occurrence for SAT-type and serotype O FMD from cattle under all three scenarios was *very low* with moderate–high uncertainty. There was a very low risk (moderate uncertainty) of SAT-type or serotype O FMDV in cattle in Kavango East or Otjozondjupa Regions, which are part of Namibia’s SAT-type FMD protection zone and not considered high risk for FMDV serotype O given their distance from Zambia. There was also a very low risk (moderate uncertainty) of cattle crossing from Namibia to Botswana through Khaudum National Park, which has a high density of predators as well as a toxic plant, *Dichapetalum cymosum,* or across Nyae Nyae Conservancy, which has few cattle near the fence (Figure 5). Similarly, the risk of SAT-type FMDV was very low (low uncertainty) in cattle in Botswana near the Western Border fence, an area which has not experienced any recent outbreaks and is under consideration for official FMD-free without vaccination status. The consequences for SAT-type and serotype O FMD are as described in the Zambezi Border fence section. The overall risk estimate for SAT-type FMD was low with moderate–high uncertainty and the risk estimate for serotype O FMD was *moderate* with moderate–high uncertainty under all three scenarios.

**Table 4:** Western Border fence risk scenarios summary of findings.

Western Border fence			
Risk scenario Disease/Route/Country	Status quo	Fence removal	Fence removal with risk mitigation
	Probability of occurrence/risk estimate		
SAT-FMD/cattle/Botswana	Very low/Low	Very low/Low	Very low/Low
SAT-FMD/buffalo/Botswana	Negligible/Low	Negligible/Low	Negligible/Low
SAT-FMD/poaching/Botswana	Negligible/Low	Negligible/Low	Negligible/Low
FMD type O/cattle/Botswana	Very low/Moderate	Very low/Moderate	Very low/Moderate
SAT-FMD/cattle/Namibia	Very low/Low	Very low/Low	Very low/Low
SAT-FMD/buffalo/Namibia	Very low/Low	Very low/Low	Very low/Low
SAT-FMD/poaching/Namibia	Negligible/Low	Negligible/Low	Negligible/Low
CBPP/cattle/Botswana	Very low or low/Moderate	Very low or low/Moderate	Very low/Moderate
PPR/small ruminants/Botswana	Very low/Low	Very low/Low	Very low/Low

Note that for SAT-type FMD outbreaks caused by a strain not covered by the vaccine, the overall risk estimate is moderate.

The probability of occurrence for SAT-type FMD from buffalo in Namibia to cattle in Botswana was *negligible* with moderate–high uncertainty under all three scenarios as the only population of buffalo in Namibia near this fence is the FMD-free herd which is in the fenced Buffalo Camp in Tsumkwe. The probability of occurrence for SAT-type FMD from buffalo in Botswana to cattle in Namibia was *very low* with moderate uncertainty, given the very low risk (moderate uncertainty) of effective contact between cattle and buffalo discussed previously.

The probability of occurrence for SAT-type FMD from poaching was *negligible* with moderate to high uncertainty under all three scenarios for reasons discussed for previous poaching pathways, with the addition of the fact that the Tsumkwe buffalo are FMD-free. With the moderate consequences (low uncertainty) of an outbreak, the overall risk estimate was *low* with moderate uncertainty.

The probability of occurrence for CBPP was *very low* with moderate uncertainty to *low* with moderate to high uncertainty under the status quo and both fence removal scenarios. This difference was based on differential risks at the steps where cattle crossed into another country: the risk of cattle crossing from Botswana to Namibia was considered low (moderate to high uncertainty), while the risk of cattle crossing from Namibia to Botswana was considered very low (moderate uncertainty). Cattle incursions have been recorded in both directions, but there are more cattle posts in Botswana near the fence than in Namibia (Figure 5) and more incursions into Namibia were reported in recent years. The risk of MmmSC in Kavango East and Otjozondjupa was also rated low, given the small number and severity of outbreaks recorded in Kavango East in the last decade (2014–3 cases, 2 deaths; 2015–20 cases, 10 deaths; 2017–16 cases, 4 deaths). The probability of occurrence for CBPP was *very low* with moderate uncertainty under the fence removal with risk mitigation scenario. Under this scenario, participation in the REHerd model by Namibian farmers would limit contact with other cattle and increase compliance with CBPP vaccination. Given the high consequences, the overall risk estimate under all three scenarios becomes *moderate* with moderate uncertainty.

The probability of occurrence for PPR was *very low* with moderate uncertainty under all three scenarios. The entry risk was very low given that there are no known sources of virus nearby. Notably this fence borders Khaudum National Park (where livestock are not permitted) and Nyae Nyae Conservancy, which has relatively small numbers of small stock (~525 animals; Lise Hanssen, unpublished data), most of which are >10 km away from the fence (Figure 5). The exposure risk was low given the relatively small numbers of goats (3–105/crush pen) recorded at the crush pens identified as high-risk in Botswana DVS’s surveillance plan. With the moderate consequences of an outbreak, the overall risk estimate was *low* with moderate uncertainty across all three scenarios.

Although some information on cattle incursions had been provided during the course of the study, both Botswana and Namibia officials believed that actual movement of livestock across the Western Border fence was higher than recorded in these official reports. Both countries agreed that the Western Border fence sections were not good candidates for potential removal at this time and recommended further data gathering on this issue. We agreed to re-evaluate the Western Border fence in the future if CBPP cases in Namibia remained sporadic and Kavango East remained free of cases, and if the countries developed a stronger bilateral understanding of illegal movement across the fence.

## 4 Discussion

### 4.1 Overarching findings

Fences have been a cornerstone of livestock disease management in southern Africa for decades (58), but land-use changes, climate change (59), and governments’ budgetary challenges have necessitated exploration of other solutions to transboundary animal disease control. Fences are not an infallible solution for disease control, as even the Kruger National Park system, which has a well-maintained western boundary fence, has seen many FMD outbreaks in cattle outside the park originating from wildlife (60–62). In the context of TFCAs, the economic implications of veterinary fences across sectors warrant consideration of more holistic approaches to ensure resilient, sustainable and thus logical beneficiation to a full range of stakeholders, especially those still living in poverty. Decision makers should take notice of the utility of recognized husbandry practices like managed herding (63), and of value chain-focused processes like CBT, to enable and expand beef market access. Fences can play an important role in disease control, but they could be applied more judiciously and cost-effectively while other risk mitigation strategies replace physical barriers in appropriate contexts.

The most striking finding from our study is that the risk level generally remained the same between the status quo and fence removal scenarios, which may seem surprising. There are several important factors which underpin these findings. First, many fence sections are semi-permeable under the status quo, given the many breakages observed during field visits, although movements of wildlife are still being impacted as observed in the Phase 1 surveys (29). Recognizing the current state of these fences is crucial in assigning risks and comparing the status quo to possible fence section removal(s). Second, we examined only select fence sections where livestock densities were relatively low, considerable fence damage already existed, and the overall perceived disease risk per the Phase 1 analysis was deemed low relative to other sections of the fences. It is important to recognize that our findings refer to removal of these specific sections only; we do not promote removal of these entire fence lines nor fence removal as a panacea, as the maps provided make clear.

Our results were also driven by the fact that fence removal impacts only some risks in the pathways. Specifically, it impacts the risk of pathogen entry; border fences do not impact the risk of a free-ranging buffalo being viremic or animal exposure risks such as the likelihood of cattle interacting with one another in their home country. It is important to note that risk mitigation measures as described, on the other hand, can lower both entry and exposure risks. Each pathway contains risk bottlenecks where the risk is negligible, very low or low, and the overall pathway risk defaults to this level based on the multiplicative nature of the combination matrix (64). Lastly, because the assessment was qualitative, each risk level represents a range rather than a numerical probability. This means that an assessment of low risk for both the status quo and the fence removal scenario may not represent the exact same risk but one which remains in the same general range.

One argument against fence removal is the risk of illegal movement of livestock and poachers. Our analysis included documentation of low levels of illegal movement already occurring with the status quo (32). The sections where fence removal is proposed have low densities of livestock (or none at all) in the surrounding areas. They are also in remote areas with many predators and terrain that is challenging even for 4×4 vehicles, areas not conducive to easy movement of livestock on foot or by vehicle. The fences likely have a limited impact on poaching, as poaching is already highly prevalent around the Okavango Delta (28) despite the presence of the buffalo fences. Importantly, our detailed assessments of FMD risk from poaching provided no support for this pathway as a likely source of FMD outbreaks in Ngamiland.

This study also draws attention to the need for re-evaluation of the emphasis on subsidized beef exports to Europe and other high-end markets, given the negative impacts on both communal farmers living in FMD-infected zones and the sustainability of the region’s wildlife populations due to fences’ ongoing impacts on critical migratory routes. Veterinary fences are largely justified by the need to protect specific zones as disease-free for export markets deemed valuable. These export markets clearly benefit farmers in green zones, but communal farmers outside the green zones have no such access to these benefits while they continue to bear the costs of living with the wildlife valued by the tourism industry. Direct partnerships with the private sector could offer new market opportunities to communal farmers; for example, local tourism operators in the Okavango Delta pay farmers using the REHerd model a premium for their “wildlife-friendly” beef to provide ration meat for staff year-round. As beef quality continues to improve, it could also be marketed with great fanfare (and good prices) to environmentally-conscious tourists. Further innovation in marketing could continue to open up more markets for wildlife-friendly (CBT-compliant) beef, and more intensive engagement of communities in the rapidly growing wildlife economy of the region is essential.

### 4.2 Northern Buffalo fence

Because the Northern Buffalo fence is an internal fence in Botswana and there are only cattle resident on one side of the fence, it presents the simplest set of disease risk pathways of all three fences and the fewest risks associated with its removal. Reopening a corridor in this area has the potential to alleviate significant human-wildlife conflict, depending on how elephants move in a reconnected landscape. Preliminary work is underway to model elephant movements under different scenarios of fence section removal and changes in seasonal water availability (65). The greatest concern about removing this fence section was the risk of increased buffalo-cattle contact for the villages in the eastern panhandle, hence the focus on improved herding as a key risk mitigator.

We note that surveys show that the Northern Buffalo fence is currently failing to keep buffalo out of the livestock rearing area, but this area has not experienced any FMD outbreaks despite the presence of buffalo near livestock along the eastern panhandle. The Phase 1 report recommended the northern section (80 km) of the Northern Buffalo fence for removal. The northern part of the fence along NG13 presents the least risk in terms of fence removal, as the only crush (Seshokora) is epidemiologically isolated from all other crushes in the event of an outbreak. Removing this section of fence would allow movement of wildlife, likely including elephants constrained by Ngamiland fences (31), out of the panhandle with no added risk of cattle contact. The southern aspect of the Northern Buffalo fence is associated with a higher risk of cattle-buffalo contact, given the number of cattle and the density of buffalo in the northern delta, although the overall risk of disease transmission from buffalo appears to be very low. Some villages in the eastern panhandle are already implementing REHerd practices, but the risk of buffalo-cattle contact and human-wildlife conflict would be further mitigated if the REHerd model were implemented on a broader scale.

### 4.3 Zambezi Border fence

The Zambezi Border fence, east of the Okavango River, presents a more complex set of risks for transboundary animal diseases. The fence section bordering Bwabwata National Park represents less risk than elsewhere along the Zambezi Border fence, given the absence of cattle in Botswana along much of it. The removal of the cattle in Bwabwata has been a key factor for Botswana DVS in terms of their consideration of removal of this fence section (47) as cattle removal would mark a major reduction in risk. The small number of cattle posts near the fence on the Botswana side would be excellent targets for implementation of REHerd practices to further protect cattle from cross-border infections in this region. The 35 km section of this fence proposed for removal represents a conservative approach to opening up this border with Namibia, but in combination with removal of the proposed 62 km section from the Northern Buffalo fence, a critical KAZA Wildlife Dispersal Area would be restored.

At the validation meeting, Botswana DVS indicated that the most significant risk at this fence was FMD serotype O; although the probability of occurrence is *very low*, the high consequences and subsequent moderate overall risk were concerning (CBPP had an identical risk profile but was not as high of a concern for Botswana DVS in this part of the country). FMDV serotype O has the potential to enter Botswana along the border with Zambezi Region, where the previous outbreak in Namibia occurred, although entry into Chobe District would arguably be more likely than Ngamiland. However, Namibia’s Directorate of Veterinary Services is now vaccinating against FMDV serotype O in Zambezi Region, sensitized to the risk in this area, making it unlikely that another outbreak of FMD serotype O would occur near the Zambian border, move unchecked into Bwabwata National Park, and spread across the border to Botswana. The crush at Seshokora also represents an epidemiologically isolated unit of cattle unlikely to spread disease elsewhere in Ngamiland if an outbreak did occur there.

It is important to note that SAT-type FMDV is endemic in free-ranging buffalo on both sides of the fence, therefore any movement of buffalo from one country to another arguably does not change the risk of the disease occurring. Similarly, there is no difference in the disease risk – which has a very low probability of occurrence – from poaching a buffalo in one country versus the other. All scenarios with buffalo and poaching near the Zambezi Border fence were considered to have a low overall risk.

### 4.4 Western Border fence

The Western Border fence represented the most complex situation and, ultimately, implications for Botswana DVS as well as some concerns related to wildlife (described below) precluded further consideration of any fence section removal at this time. Entry of MmmSC is without question the greatest concern at this fence line based on the severity of consequences from the last CBPP outbreak. Although the current probability of occurrence for CBPP was very low or low, the risk here is arguably higher than at the Zambezi Border fence, given the closer proximity to recent outbreaks and regions where cattle are routinely taken to Angola for grazing. Incursion reports suggest that livestock movement is greater across this fence than the others considered in our study, and the true level of livestock movement across this fence is likely greater than the reported incursions.

The Western Border fence has negligible FMDV risk to Botswana associated with buffalo, as the only buffalo nearby in Namibia are FMD-free. There are no or limited resident buffalo in Botswana near the fence, although dispersing animals from Botswana do occasionally move across the border (66). This is problematic in that dispersing buffalo will be naturally infected with SAT-type FMDV while the only buffalo in Namibia’s protection zone west of Zambezi Region are those in Tsumkwe, all of which are FMD-free (and notably, fenced-in). Plans for wildlife reintroductions into Khaudum National Park also need to be carefully considered in decision-making about this fence. The park’s management plan states that buffalo as well as white rhinoceros (*Ceratotherium simum*) and black rhinoceros (*Diceros bicornis*) may be established in the park. If the buffalo population in the park were to be FMD-free, then cattle and buffalo incursions from Botswana could jeopardize the FMD-free status of such animals. The threat of poaching for rhino horn also makes border security for the park an utmost priority.

### 4.5 Limitations

This analysis has a number of limitations. Our study did not involve direct engagement of local communities, which we recognize is an important component of risk communication and equitable decision-making regarding fencing policies. Local community dynamics are particularly important in our region, where extended families or ethnic groups may be physically divided by fences and cross-border contact or trade may be regular. Community engagement was always envisaged as a separate and final (Phase 3) component of this multi-phase project, to be led by a social science team with engagement targeted specifically at communities in areas with fence sections that were agreed upon as potential candidates for removal by government officials. In fact, at the time of this writing, such community consultations have begun. Gauging community perceptions of the pros and cons of the fences (historically and today) and understanding communities’ level of interest in participating in proposed risk mitigation measures like REHerd is not only crucial to any future decisions, but would have better approximated uncertainty levels during the risk analysis study itself.

Botswana and Namibia are part of the greater KAZA landscape, and neighbor Zambia and Angola where CBPP exists. Including data from the latter countries was beyond the scope of our assessment; we acknowledge there is risk from movements of animals from Angola and Zambia. We also note that there are other transboundary animal diseases which we did not explore in our study but which have the potential to be transmitted between countries.

Other factors in our study region make it difficult to fully capture the dynamic risks of these livestock diseases. The traditional practice of transhumance means that cattle are mobile across communal lands to varying degrees rather than restricted to a specific area. Artificial water provisioning is another important issue affecting animals’ movements. In the eastern panhandle, there are boreholes in operation, with the rights for many others already allocated but currently unused. Drilling of new boreholes for livestock or wildlife will inherently affect animal movements and draw animals to areas where they do not occur in high densities currently, with the potential for new areas of livestock-wildlife interaction and/or human-wildlife conflict. Collaborative planning of grazing over time and space, as emphasized through the REHerd program, would be beneficial to ensure that livestock have access to adequate water and grazing throughout the year, whilst mitigating key risk factors.

The qualitative nature of the assessment means that there is some inherent uncertainty in our results; exact risk calculations are simply not possible given the data available. Nonetheless, we believe that our qualitative assessment, supplemented with a multilateral validation workshop process to address subtleties that cannot be adequately captured in risk matrices, provides a strong, evidence-based mechanism to guide decision-making.

### 4.6 Conclusion

The outcomes from this assessment signal the potential for a new paradigm in veterinary disease control in southern Africa. Current funding and staffing levels in departments of veterinary services are failing to support the continuous maintenance of existing veterinary fences in the face of ongoing damage from elephants, flooding and other wear and tear. Veterinary fences as historically envisioned and advanced are also now in direct conflict with the wildlife habitat connectivity goals critical to the ecological and economic success of a TFCA like KAZA (22). Recent work by Hauptfleisch et al. (67) in Namibia also suggested reconsideration of veterinary fences depending on land-use opportunities, noting the potential benefits of wildlife utilization. Our study further highlights the potential role strategic herding and kraaling practices, especially if done by skilled herders, could have in reducing risk of disease transmission at a landscape level. We believe our study is the first to use a risk assessment framework to support science-based decision-making on existing disease control fencing.

Removing sections of the Zambezi Border and Northern Buffalo fences as proposed here would open the eastern panhandle to the rest of the Kwando River Wildlife Dispersal Area, potentially alleviating elephant population pressure and conflict in the panhandle, in addition to bringing other benefits to wildlife and the regional economy. Such restoration of KAZA’s connectivity will be increasingly vital given climate change’s impacts on the availability of grazing and water resources over space and time. Restoration of wildlife movement patterns will be impacted by complex interactions among ecological communities, climate change, hydrological cycles, availability of preferred habitat or prey species, expanding human settlements, agriculture and infrastructure developments, and other factors. The community consultations that comprise Phase 3 are a critical next step in KAZA partner states’ ongoing efforts to assess the feasibility of removing these fence sections and developing a new, more sustainable approach to transboundary animal disease management in a One Health context.

## Data Availability

Publicly available datasets were analyzed in this study. This manuscript is derived from the original report produced by the KAZA fencing scenarios disease risk assessment project, which is published on the Cornell AHEAD Program’s website (https://www.wcs-ahead.org/documents/livestock-dz-risk-assess_lo_res_final_20240917.pdf).
