# Supplementary material for: Using qualitative risk assessment to re-evaluate the veterinary fence paradigm within the Kavango Zambezi Transfrontier Conservation Area
Source: Front Vet Sci. 2026 Jan 30;12:1702631. doi: 10.3389/fvets.2025.1702631 (PMC12903917; doi:10.3389/fvets.2025.1702631)
Supplement: Supplementary file 1 [file Table_1.docx]

Supplementary Material

# Supplementary Tables

**Supplementary Table 1.** Probability risk levels used for the entry and exposure assessments, adapted from Rinchen et al. (1).

| **Probability** | **Definition** |
| --- | --- |
| Negligible | Likelihood of an event occurring is so rare that it does not merit consideration |
| Very low | Likelihood of an event occurring is rare but can occur |
| Low | Likelihood of an event occurring is occasional |
| Moderate | Likelihood of an event occurring is regular |
| High | Likelihood of an event occurring is very often |

**Supplementary Table 2.** Uncertainty categories used in the assessment, as defined by Fournié et al. (2).

| **Uncertainty Category** | **Definition** |
| --- | --- |
| Low | There are solid and complete data available; strong evidence is provided in multiple references; authors report similar conclusions. Several experts have multiple experiences of the event, and there is a high level of agreement between experts. |
| Moderate | There are some but not complete data available; evidence is provided in a small number of references; authors report conclusions that vary from one another. Experts have limited experience of the event and/or there is a moderate level of agreement between experts. |
| High | There are scarce or no data available; evidence is not provided in references but rather in unpublished reports or based on observations, or personal communication; authors report conclusions that vary considerably between them. Very few experts have experience of the event and/or there is a very low level of agreement between experts. |

**Supplementary Table 3.** Consequence levels and definitions used in the assessment, as defined by Zepeda-Sein (3).

| **Magnitude** | **Definition** |
| --- | --- |
| Negligible | Little or no impact on production, production costs, health and longevity of the hosts, national and international sales |
| Low | Minor impact on the factors above |
| Moderate | Impact of medium magnitude on the factors above |
| High | Severe impact on the factors above |

**Supplementary Table 4.** Combination matrix used for combining two probabilities, modified from Rinchen et al. (1).

|  | **Probability 1** | | | | |
| --- | --- | --- | --- | --- | --- |
| **Probability 2** | **Negligible** | **Very Low** | **Low** | **Moderate** | **High** |
| **Negligible** | Negligible | Negligible | Negligible | Negligible | Negligible |
| **Very low** | Negligible | Very low | Very low | Very low | Very low |
| **Low** | Negligible | Very low | Low | Low | Low |
| **Moderate** | Negligible | Very low | Low | Moderate | Moderate |
| **High** | Negligible | Very low | Low | Moderate | High |

**Supplementary Table 5**. Risk estimation matrix used for combining probability of occurrence and consequences, modified from Dufour et al. (4).

|  | **Probability of Occurrence** | | | | |
| --- | --- | --- | --- | --- | --- |
| **Consequences** | **Negligible** | **Very Low** | **Low** | **Moderate** | **High** |
| **Negligible** | Negligible | Negligible | Negligible | Negligible | Negligible |
| **Very low** | Negligible | Negligible | Negligible | Very low | Very low |
| **Low** | Negligible | Very low | Very low | Very low | Low |
| **Moderate** | Low | Low | Low | Moderate | Moderate |
| **High** | Moderate | Moderate | Moderate | Moderate | High |

Supplementary References:

1. Rinchen S, Tenzin T, Hall D, Cork S. A qualitative risk assessment of rabies reintroduction into the rabies low-risk zone of Bhutan. *Front Vet Sci* (2020) 7:366. doi: 10.3389/fvets.2020.00366

2. Fournié G, Jones BA, Beauvais W, Lubroth J, Njeumi F, Cameron A, Pfeiffer DU. The risk of rinderpest re-introduction in post-eradication era. *Prev Vet Med* (2014) 113:175–184. doi: 10.1016/j.prevetmed.2013.11.001

3. Zepeda-Sein C. Méthodes d’évaluation des risques zoosanitaires lors des échanges internationaux. *Séminaire sur la sécurité zoosanitaire des échanges dans les Caraıbes*. Port of Spain, Trinidad: Office International des Epizooties (1998). p. 61–76

4. Dufour B, Plee L, Moutou F, Boisseleau D, Chartier C, Durand B, Ganiere JP, Guillotin J, Lancelot R, Saegerman C, et al. A qualitative risk assessment methodology for scientific expert panels. *Rev sci tech Off int Epiz* (2011) 30:673–681. doi: 10.20506/rst.30.3.2063
